# Genetic variations in the beta-tubulin gene and the internal transcribed spacer 2 region of *Trichuris* species from man and baboons

**DOI:** 10.1186/1756-3305-6-236

**Published:** 2013-08-12

**Authors:** Tina VA Hansen, Stig M Thamsborg, Annette Olsen, Roger K Prichard, Peter Nejsum

**Affiliations:** 1 Department of Veterinary Disease Biology, Faculty of Health and Medical Sciences, University of Copenhagen, Dyrlægevej 100, DK-1870 Frederiksberg C, Denmark; 2 Institute of Parasitology, McGill University, Ste. Anne-de-Bellevue, Canada

**Keywords:** *Trichuris trichiura*, Nematode, Beta-tubulin, Anthelmintic resistance, SNPs, ITS2, Baboon, Human

## Abstract

**Background:**

The whipworm *Trichuris trichiura* has been estimated to infect 604 – 795 million people worldwide. The current control strategy against trichuriasis using the benzimidazoles (BZs) albendazole (400 mg) or mebendazole (500 mg) as single-dose treatment is not satisfactory. The occurrence of single nucleotide polymorphisms (SNPs) in codons 167, 198 or 200 of the beta-tubulin gene has been reported to convey BZ-resistance in intestinal nematodes of veterinary importance. It was hypothesised that the low susceptibility of *T. trichiura* to BZ could be due to a natural occurrence of such SNPs. The aim of this study was to investigate whether these SNPs were present in the beta-tubulin gene of *Trichuris* spp. from humans and baboons. As a secondary objective, the degree of identity between *T. trichiura* from humans and *Trichuris* spp. from baboons was evaluated based on the beta-tubulin gene and the internal transcribed spacer 2 region (ITS2).

**Methods:**

Nucleotide sequences of the beta-tubulin gene were generated by PCR using degenerate primers, specific primers and DNA from worms and eggs of *T. trichiura* and worms of *Trichuris* spp. from baboons. The ITS2 region was amplified using adult *Trichuris* spp. from baboons. PCR products were sequenced and analysed. The beta-tubulin fragments were studied for SNPs in codons 167, 198 or 200 and the ITS2 amplicons were compared with GenBank records of *T. trichiura*.

**Results:**

No SNPs in codons 167, 198 or 200 were identified in any of the analysed *Trichuris* spp. from humans and baboons. Based on the ITS2 region, the similarity between *Trichuris* spp*.* from baboons and GenBank records of *T. trichiura* was found to be 98 – 99%.

**Conclusions:**

Single nucleotide polymorphisms in codon 167, 198 and 200, known to confer BZ-resistance in other nematodes, were absent in the studied material. This study does not provide data that could explain previous reports of poor BZ treatment efficacy in terms of polymorphism in these codons of beta-tubulin. Based on a fragment of the beta-tubulin gene and the ITS2 region sequenced, it was found that *T. trichiura* from humans and *Trichuris* spp. isolated from baboons are closely related and may be the same species.

## Background

The human whipworm, *Trichuris trichiura,* has been estimated to infect 604 – 795 million people worldwide [1] resulting in an expected 6.4 million disability adjusted life-years lost globally [2].

The current main control strategy against *T. trichiura*, and the other soil-transmitted helminths (STHs) (*Ascaris lumbricoides*, *Necator americanus* and *Ancylostoma duodenale*), is administration of single-dose anthelmintics drugs. The benzimidazoles (BZs), albendazole (ALB) and mebendazole (MBD) are the most widely used anthelmintics in large-scale control programs. They are administered, primarily to school-aged children, at a single dosage of 400 mg and 500 mg, respectively [3]. However, the efficacy of this single dose strategy is not satisfactory against *T. trichiura*. A meta-analysis of 20 randomized, placebo-controlled trials reported an average cure rate (CR) of 28% for ALB (400 mg) and 36% for MBD (500 mg) [4]. Other randomized controlled trials have reported similar low efficacies, with CRs ranging from 31.5% to 40.3% and egg reduction rates (ERR) from 9.8% to 54% for ALB. For MBD, CRs between 22.9% to 66.7% and ERRs from 18.8% to 81% have been found [5-7].

The use of single-dose treatment as well as multiple-dose treatment is associated with low efficacy in animals infected with *Trichuris* spp. Low to varied efficacy of both pro-BZs (i.e. netobimin and febantel) and BZs (e.g. thiabendazole (TBZ), fenbendazole (FBZ) and MBD) have been reported in different animal species [8-13]. The explanation for this low and varied efficacy of BZs against *Trichuris* spp. infection in both man and animals is not known.

The anthelmintic effect of BZs is related to its binding to beta-tubulin and the subsequent prevention of microtubule polymerization, causing destabilization of the intracellular environment and inhibition of cell division in the parasite [14]. Genetic changes in the beta-tubulin gene have been reported to convey BZ-resistance in *Caenorhabditis elegans*[15] and several parasitic nematodes. The most commonly described genetic changes are single nucleotide polymorphisms (SNPs) in the beta-tubulin gene isotype 1. These mutations are found in codon 200, 167 (both TTC to TAC) or codon 198 (GAA to GCA) and cause specific amino acid substitutions. In codons 200 and 167, these changes lead to tyrosine substituting for phenylalanine (Phe200Tyr and Phe167Tyr), and a change in codon 198 causes glutamate to be changed to alanine (Glu198Ala) [16]. SNPs in these codons have been found in several BZ-resistant nematodes of veterinary importance [17-22]. Interestingly, a SNP in codon 200 has recently been identified in *T. trichiura* obtained from a human population expected to be unexposed to BZs, suggesting that it is naturally occurring in this helminth species [23]. In addition to these SNPs in isotype 1 of the beta-tubulin gene, genetic changes in isotype 2 or loss of individuals with isotype 2 have been associated with resistance to BZs in *Haemonchus contortus*[24,25]. In *T. trichiura* only a single beta-tubulin isotype has been identified [26].

Based on morphology and supported by phylogenetic findings, *Trichuris* spp. infecting non-human primates have been reported to be closely related or identical to *T. trichiura*[27-30]. Ravasi *et al.*[30] found that *Trichuris* spp. obtained from chacma baboons (*Papio ursinus)* shared 98 – 99% identity with *T. trichiura* isolated from a human patient in China based on the internal transcribed spacers (ITS) of ribosomal DNA (ITS1-5.8S-ITS2). Interestingly, another distinct cluster containing worms obtained from another human, and from baboons, was identified by phylogenetic analysis based on the ITS region [30]. These results suggest that two or multiple *Trichuris* genotypes are infecting the two host species and that man and baboons possibly share the same *Trichuris* species.

The aim of this study was to investigate whether SNPs in the beta-tubulin gene, known to be associated with BZ-resistance in nematodes, occurred in *T. trichiura* samples from humans and *Trichuris* spp. isolated from baboons. A second objective was to determine the genetic relationship between *Trichuris* spp. obtained from the two host species using the beta-tubulin gene and the ITS2 region of some of the baboon worms. This was done in order to assess whether the two hosts share the same *Trichuris* species and to evaluate the baboon as a future host model for *T. trichiura* infections.

## Methods

### Parasite material

Adult stages (*n* = 27) of *T. trichiura* were collected from stool samples from 17 humans in Uganda (UG) after treatment with 100 mg MBD 2 × daily for 5 days [31]. One worm with unknown history from a human patient in China was also included. A total of 49 adult stages of *Trichiura* spp. were recovered from baboons at necropsy. The baboons were euthanized due to management reasons and not issues related to this project. From Denmark (DK), 23 worms were recovered from 3 hamadryas baboons (*Papio hamadryas*) in Copenhagen Zoo and 21 worms from 2 hamadryas baboons in Knuthenborg Safari Park. From United States (US), 5 worms were obtained from 3 olive baboons (*Papio hamadryas anubis*) in Southwest National Primate Research Center. The treatment history of the hamadryas baboons from the Copenhagen Zoo was either moxidectin, ivermectin or fenbendazole given twice per year, administered in the feed whereas the treatment history of the baboons from Knuthenborg Safari Park was unknown. Baboons in Southwest National Primate Research Center are injected with ivermectin (1%) twice a year. None of the animals were under anthelmintic treatment when the worms were recovered. Recovered parasites were washed in tap water and stored in 70% ethanol at 5°C until further analysis. *T. trichiura* eggs obtained from humans in UG were isolated from faeces by wet sieving and embryonated in 1 M H_2_SO_4_ (pH 1) at 22°C in 40 ml culture flasks for 2 – 3 month. A total of 39 individual eggs from 7 humans, possibly exposed to anthelmintic (MBD or ALB) twice a year as part of a mass drug administration programme in the community, were analysed. Adult *T. trichiura* worms included in this study had previously been evaluated morphometrically and genetically and were found to be distinguishable from *T. suis*[32]*.* Morphological comparison of *T. trichiura* from the humans and *Trichuris* derived from baboons has been made and both sets of samples appeared morphologically to be *T. trichiura*[28]. However, recent genetic studies show conflicting results in relation to *Trichuris* spp. infecting non-human primates [30,33]. Therefore, the reliability of determining *Trichuris* at species level by morphology and morphometric analysis is still debatable. For clarity, *Trichuris* isolated from humans will be referred to as *T. trichiura* and *Trichuris* from baboon as *Trichuris* spp. All parasitic materials were characterized as *Trichuris* spp. according to Taylor *et al.*[34] and Roberts *et al*. [35].

### DNA extraction

Whole male worms were used for DNA extraction whereas only the anterior part of the female worms were used in order to avoid DNA contamination from males (sperm or fertilized eggs). The DNA extraction was performed using the MasterPure DNA Purification Kit (Epicentre) according to the manufacturer’s protocol except that worm tissue was homogenized with a pestle and digested with 10 μl proteinase K (50 μg/μl) in 290 μl Tissue and Cell Lysis Solution for 12 hours. The purity and concentration of the DNA was evaluated using a NanoDrop ND-1000 (Thermo Scientific). DNA from eggs was made accessible for PCR by crushing single eggs with a needle according to Carlsgart *et al*. [36]. Disruption of the eggs was confirmed by microscopy.

### Amplification of a beta-tubulin gene fragment and purification of PCR product

The degenerate primers (beta-DF2: aaYtgggcKaaRggScacta and beta-DR1: gWggatcacaagcWgccatc) and PCR conditions described by Hansen *et al.*[37] were used to amplify a fragment of the beta-tubulin gene including codons 167, 198 and 200 from human and baboon derived *Trichuris*. The PCR products were sequenced (see below) and Primer3 was used to design more specific primers (beta-HB-F: tgcttgatgtagtccgcaag and beta-HB-R: gcaaagccaggcataaagaa) targeting conserved regions in the human and baboon *Trichuris* beta-tubulin gene. This was done in order to improve sequence quality and these specific primers were therefore subsequently used.

The cycling conditions for the PCR were as follows: 15 min at 95°C followed by 35 cycles at 95°C for 30 s, 56°C for 40 s 72°C for 1 min. and a final extension at 72°C for 10 min. Standard PCR conditions with 15 mM MgCl_2_ were used. For single eggs, the amplification was performed individually by adding PCR master mix directly to the crushed eggs. Negative water controls were included in all steps. The size of the amplicons were ~ 500 bp as expected when confirmed by gel electrophoresis on a 1.5% agarose gel (TAE, 0.5%) stained with ethidium bromide (EtBr).

For purification of the PCR products 30 U Exonuclease I (Fermentas) and FastAP Thermosensitive Alkaline Phosphatase (5 U) (Fermentas) were used per 15 μL of each PCR products. The mixture was incubated at 37°C for 15 min followed by enzyme deactivating at 85°C for 15 min.

### PCR of the ITS–2 region

The ITS2 region of 10 adult *Trichuris* spp. obtained from both hamadryas baboons and olive baboons were PCR amplified according to Nissen *et al*. [32]. The PCR products were purified as described above and direct sequencing applied (see below).

### Sequence analysis

All PCR products were sequenced in both directions by Macrogen (Seoul, Korea). Sequences were evaluated and heterozygotes identified using chromatograms in Vector NTI. All sequences were aligned using ClustalW2 [38] applying default settings and trimmed using BioEdit [39].

### Cluster analysis

The nucleotide diversity (π) was estimated with the Jukes and Cantor (JC) correction using DnaSP 5.10. [40]. Nucleotide diversity measures the average number of nucleotide substitutions per site between two sequences and JC corrects for the likelihood of multiple hits due to a high level of variation.

Arlequin 3.5.1.2 [41] was used to perform analyses of molecular variance (AMOVA) to estimate the partitioning of genetic variation within and between populations. The fixation index, Fst [42] was estimated and significant differentiation between populations was computed using 1023 permutations.

Molecular Evolutionary Genetic Analysis (MEGA) version 5.05 [43] was used for constructing dendrograms based on the beta-tubulin gene. JC was used as distance model and Maximum Likelihood (ML), Neighbour-joining (NJ), Minimum Evolution (ME) and Unweighted pair-group method with arithmetic means (UPGMA) applying default settings were used for constructing dendrograms. For ME the ‘nearest neighbour interchange distance’ was used to compare distances between trees and used in heuristic search. The dendrograms were rooted with beta-tubulin sequences from *Trichuris* isolated from pig, mouse and dog [37] and stability of dendrogram topology was evaluated using bootstrap with 1000 replications. Dendrograms were compared by visual inspection.

### Ethical considerations

Ethical approval was obtained from subjects receiving MBD and donating *T. trichiura* worms. Parents and children were informed about the study. The children received a consent form in both English and the local language for their principal caretaker to sign. Only those children who were willing, and where the caretaker consented, participated in the study.

## Results

### Beta-tubulin polymorphic sites and analysis of codons 167, 198 and 200

**Figure 1:**
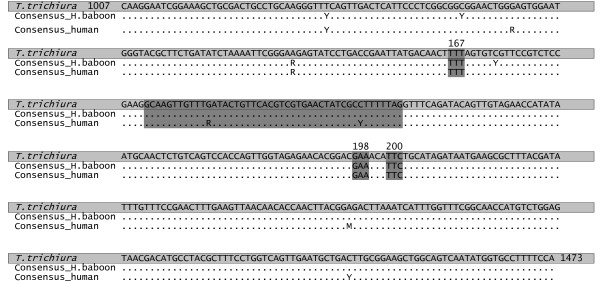
**Consensus sequences of a 467 bp beta-tubulin fragments, including codon 167, 198 and 200.** Consensus sequences are from *Trichuris* spp. specimens obtained from humans and baboons. A *T. trichiura* sequence from GenBank (AF034219) is included in the figure (light grey). Codons 167, 198 and 200 are highlighted in dark grey as well as the intron spanning 46 bp. Abbreviations for variable sites and heterozygotes: *Y* C/T, *R* A/G, *M* A/C.

Consensus sequences of a 467 bp beta-tubulin fragment generated for *Trichuris* spp. samples from humans and baboons are given in Figure 1. The sequences include codon 167, 198 and 200 which are highlighted in grey together with an intron spanning 46 bp. The nucleotide numbers (1007 and 1473) refer to the full sequence of the beta-tubulin gDNA from *T. trichiura* ([GenBank: AF034219], full length: 2482 bp), which is also included in Figure 1.

No polymorphism in codons 167, 198 or 200 was found in the 27 *T. trichiura* worms and 39 individual eggs obtained from humans and 49 *Trichuris* spp. worms obtained from hamadryas- and olive baboons. A total of 34 polymorphic sites were found in the coding part of the beta-tubulin fragment, of which 33 were synonymous and 1 non-synonymous. The non-synonymous mutation was found at bp 1102 and resulted in an amino acid change from serine to tyrosine.

### Genetic diversity and cluster analysis

The nucleotide diversity (π) of the 467 bp fragment of the beta-tubulin gene is given in Table 1 for exon, intron and the overall fragment. The nucleotide diversity within exon, intron and the overall fragment was higher in *T. trichiura* from humans than *Trichuris* spp. isolated from baboons.

**Table 1:** **Nucleotide diversity (π) in a 467 bp fragment of the beta-tubulin gene from *Trichuris *isolated from humans and baboons**

		**Human**	**Baboon**
**Exon**	**Human**	0.003	
**Intron**	*n = 68*	0.009	
**Overall**		0.004	
**Exon**	**Baboon**	*0.004*	0.002
**Intron**	*n* = 49	*0.005*	0.000
**Overall**		*0.004*	0.002

The nucleotide diversity in exon, intron and the overall fragment are shown as well as the nucleotide diversity within and between (italic) hosts.

Heterozygosity within individual adult stages and eggs was found at 9 nucleotide positions and is given in Figure 1. Heterozygosity at two of these nucleotide positions (1067 and 1151) were found in 2 worms from hamadryas baboons in Copenhagen Zoo and 2 worms from hamadryas baboons in Knuthenborg Safari Park. Heterozygosity in 5 out of the 9 nucleotide positions (1076, 1178, 1205, 1359 and 1437) was found only in *T. trichiura* from Uganda in both adult worms and eggs. Heterozygosity at nucleotide positions 1043 and 1115 was found in both *T. trichiura* isolated from humans and *Trichuris* spp. isolated from baboons.

There was no genetic differentiation between the population of worms and eggs obtained from humans in Uganda (Fst = 0.02, *P* = 0.14) or *Trichuris* spp. worms obtained from baboons in the Copenhagen Zoo and Knuthenborg Safari Park (Fst = 0.03, *P* = 0.76). In contrast, high and significant population differentiation between the Danish baboon worms and *T. trichiura* obtained from humans in Uganda (Fst = 0.38, *P* < 0.001) was found.

**Figure 2:**
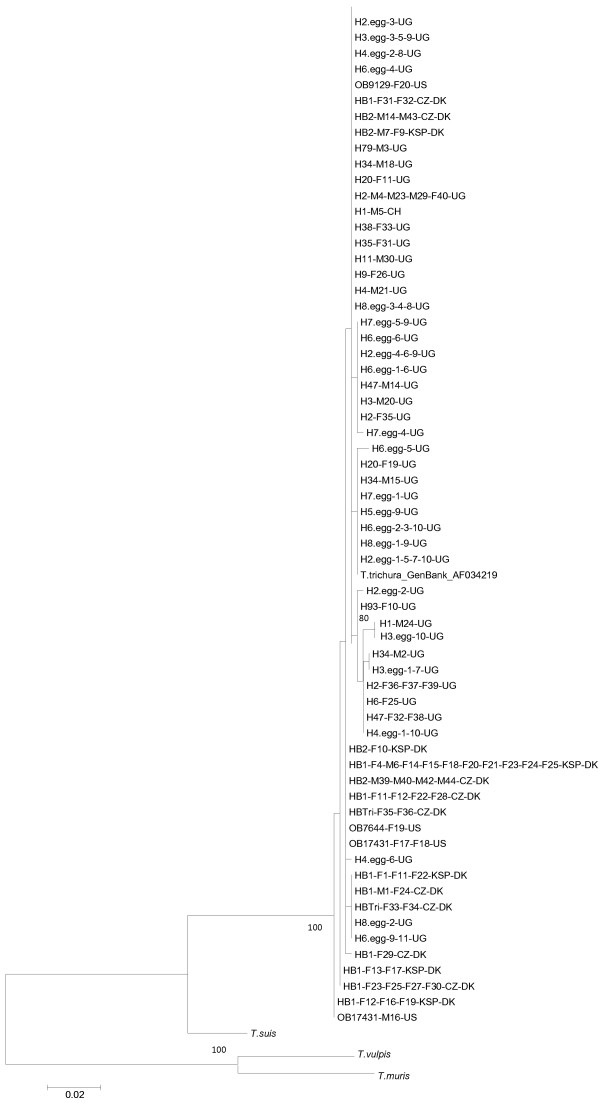
**Maximum likelihood tree based on sequences (467 bp) of the beta-tubulin gene.** The tree shows the genetic relationship between *Trichuris* spp. isolated from humans (eggs and worms) and baboons (worms) as well as *T. trichiura* [GenBank:AF034219]. The tree was rooted with *Trichuris* isolated from pig, mouse and dog [37]. Bootstrap values above 80 are reported. Identification of individual worms and eggs are listed after host identification and merged with (−) when obtained from the same host and segregating into the same clade. Scale bar: number of base substitutions per site. Abbreviations for host species, parasite gender and origin of parasitic material: H: human; HB: hamadryas baboon; OB: Olive baboon, F: female; M: male; KSP: Knuthenborg Safari Park; CZ: Copenhagen Zoo. The final two letters indicate the geographic origins (DK, Denmark; UG, Uganda; CH, China; US, United States).

The genetic relationship between *T. trichiura* from humans and *Trichuris* spp. isolated from baboons was evaluated by 4 different cluster methods. All methods resulted in identical tree topology; ML is given in Figure 2. Beta-tubulin sequences generated from *T. trichiura* and *Trichuris* spp. isolated from baboons did not cluster together in clades according to host or geographical origin, but were interspersed with each other in the tree. A total of twelve 467 bp beta-tubulin sequences from *Trichuris* isolated from baboons [GenBank:KF410623-KF410628] and humans [GenBank:KF410629-KF410634] are available in GenBank.

### The ITS2 sequence from hamadryas- and olive baboons

The ITS2 region of 10 *Trichuris* spp. worms, obtained from baboons, was amplified but only 4 amplicons could be sequenced (1 from a single hamadryas baboon in Copenhagen Zoo, DK and 1 each from three olive baboons in the Southwest National Primate Research Center, US). The length variation in the ITS2 sequences was 478 – 520 bp. A Blast search was conducted in GenBank and three out of 4 sequences, from both Denmark and United States, had 99% identity with *T. trichiura* isolated from humans in Uganda [GenBank:JN181849, JN181850] and 98 – 99% identity with *Trichuris* spp. isolated from baboons living in South Africa [GenBank:GQ301551, GQ301552, GQ301553]. The fourth sequence had 98% and 96% identity with *T. trichiura* isolated from humans in Uganda [GenBank: JN1818543, JN181857, JN181859] and *Trichuris* spp. isolated from the above described baboons. The four ITS2 sequences from baboons are deposited in GenBank: [GenBank:KF410635] (*Trichuris* spp. from *P. h. anubis/P. h. cynocephalus,* US), [GenBank:KF410636] (*Trichuris* spp. from *P. hamadryas*, DK), [GenBank:KF410637, KF410638] (*Trichuris* spp. from *P. h. anubis*, US).

## Discussion

None of the SNPs in codons 167, 198 or 200 of the beta-tubulin gene, previously found to be associated with BZ-resistance in parasitic nematodes, was observed in any of the 27 adult worms or 39 eggs of *T. trichiura* from humans or the 49 adult *Trichuris* spp. isolated from baboons. This is in contrast to a previous study in which a SNP in codon 200 was found in some *T. trichiura* isolated from school children in Kisumu, Kenya, who themselves had not been treated with BZ anthelmintics, although there may well have been previous treatments in the community. Forty-one per cent of these whipworms were found to be heterozygotes (TAC/TTC) and 2.6% homozygotes (TAC/TAC) [23]. In the present study all of the analysed *T. trichiura* worm samples were expelled from humans in Uganda, following MBD treatment and were presumably BZ susceptible, except for one worm from China in which the treatment history was not known. As *T. trichiura* has been shown to be genetically differentiated between countries [44], this and differences in community treatment and methods of collection may explain why similar frequencies of these SNPs were not observed between the two studies. It is not known whether BZ-resistance will be recessive, dominant or semi-dominant in *Trichuris* spp., but based on SNPs in codon 200 in other parasitic nematodes, BZ-resistance is likely to be a recessive trait [44]. Therefore, one would not expect to find any homozygotes (TAC/TAC) in the adult *T. trichiura* recovered from humans following MBD treatment. However, the eggs of *T. trichiura* were obtained from humans not treated with any anthelmintic. These eggs were included to examine any potential influence from the inclusion of worms obtained after “purgation” with MBD, as such worms were likely to be BZ susceptible. The likelihood of observing SNPs in the eggs was, therefore, higher than in the adult worm material.

Variation in codons 167 and 198 of the beta-tubulin gene has previously been reported for *Trichuris* spp. isolated from a range of wild and domesticated animals [37]. However, in the present study no differences in codons 167, 198 or 200 between *T. trichiura* from humans and *Trichuris* spp. isolated from baboons were observed, and the nucleotides in these codons were found to be the same as reported in *T. suis* isolated from pigs [37].

The nucleotide diversity in the exon, intron and the overall fragment of *T. trichiura* from humans and *Trichuris* spp. isolated from baboons were 0.003; 0.009; 0.004 and 0.002; 0.00; 0.002, respectively. The low diversity is in close agreement with Bennett *et al.*[45], who found a nucleotide diversity between 0.001 – 0.005 in the overall fragment (1079 bp) of the beta-tubulin gene and between 0.00 – 0.01 in the intron among *T. trichiura* from various geographical locations. In addition, *Trichuris* spp. from both domestic animals and wildlife have been reported to have low nucleotide diversity in the beta-tubulin gene [37]. In contrast to the low nucleotide diversity found in this and the above mentioned studies, the nucleotide diversity in parasitic nematodes of veterinary importance has been reported to be higher. In BZ-susceptible strains of *H. contortus* the nucleotide diversity was found to be 0.094 and 0.091 in a fragment of isotype 1 (1600 bp) and isotype 2 (1450 bp), of the beta–tubulin gene when evaluated by restriction fragment length polymorphism (RFLP) [24]. In BZ-resistant strains of *Teladorsagia circumcincta* a nucleotide diversity of 0.06 was found in a 276 bp fragment of isotype 1 beta-tubulin covering codons 167, 198 and 200 [46]. The genetic diversity was found to be higher in *T. trichiura* from humans than for *Trichuris* spp. isolated from baboons in particular in the intron (human: 0.009; baboon: 0.000) probably because the baboon population has been through a genetic bottleneck as the animals were kept in captivity. A high genetic diversity would increase the possibility that resistant alleles would be present in a population of parasitic nematodes [47].

Despite the fact that *Trichuris* is highly prevalent among baboons, their taxonomic status is unsettled due to lack of discrete morphological criteria. However, they are often designated as *T. trichiura* as they are expected to be the same species as the one found in humans [28,48]. The cluster analysis based on the beta-tubulin gene (Figure 2) supports this assumption that humans and baboons share the same *Trichuris* species as worms from the two hosts are interspersed in the tree. This is further supported by ITS2 sequences from 4 baboon worms as they shared 98 – 99% identity with *T. trichiura* isolated from humans in Uganda. The high identity found between *Trichuris* isolated from baboons and humans is in concordance with Ravasi *et al.*[30] who, based on ITS1-5.8S-ITS2 sequences, found 98 – 99% identity between 3 *Trichuris* spp. isolated from chacma baboons in South Africa and *T. trichiura* from a patient in China [GenBank:AM992981]. In the present work it was only possible to sequence the ITS2 region in 4 out of 10 *Trichuris* spp. worms, which was probably due to variable number of tandem repeats (intra-individual length variation). In future work, this could be addressed using cloning-techniques prior to sequencing. Elucidating the taxonomic relationship between *Trichuris* infecting humans and baboons, in relation to unsatisfactory anthelmintic efficacy, is highly relevant for validating the baboon as a future model for human trichuriasis.

In the few efficacy studies of BZ in baboons, FBZ was applied either in a triple dosage regime (50 mg/kg for 3 consecutive days) [49] or offered in a commercial primate diet formulated with FBZ at 600 mg/kg concentrate [50]. In both studies the efficacies were relatively high: 96.3 – 99.1% reduction in faecal egg counts with triple dosage treatment and 92.6 – 100% when administered in the diet. However, the efficacy of FBZ in these studies is not comparable with the efficacy of ALB and MBD in human trichuriasis. Firstly, the dosages applied with the triple dosage regime exceeded the dosages of ALB (400 mg) or MBD (500 mg) used in human trichuriasis (i.e. 760 – 1675 mg FBZ daily). Secondly, a 3-days dosage regime was used or the exact doses were unknown due to the administration method. Lastly, although ALB, MBD and FBZ all belongs to the BZ group their pharmacokinetic properties depend on many factors such as variation within and between species, drug formulation and route of administration [51,52] which could impact relative efficacy.

## Conclusion

Based on the analysis of the beta-tubulin gene, it was found that SNPs known to confer BZ-resistance in other nematodes, were absent in the analysed *Trichuris* samples isolated from humans and baboons. Based on both a fragment of the beta-tubulin gene and the ITS2 region, it was found that *T. trichiura* from humans and *Trichuris* spp. isolated from baboons were closely related and perhaps identical. The explanation for a low to varied efficacy of BZs against *Trichuris* spp. infections in both man and animals is yet unknown. Since *Trichuris* spp. infecting baboons are identical or closely related to *T. trichiura* and a similar genome organization exists between the baboon (*Papio hamadryas*) and man [53]*,* a baboon model could be useful in elucidating the causes of an unsatisfactory efficacy of single-dose BZs against *T. trichiura* infection in humans and to determine how efficacy could be improved.

## Competing interests

The authors declare that they have no competing interest.

## Authors’ contributions

All authors contributed equally in planning the study. TVAH carried out the experiments, the data analysis and wrote the manuscript. PN participated in the experiments, the data analysis and in writing the manuscript. SMT and AO participated in data analysis and performed critical revisions of manuscript drafts. RKP performed critical revisions of manuscript drafts. All authors read and approved the final manuscript.
